# Supplementary material for: Identifying Drug Response by Combining Measurements of the Membrane Potential, the Cytosolic Calcium Concentration, and the Extracellular Potential in Microphysiological Systems
Source: Front Pharmacol. 2021 Feb 8;11:569489. doi: 10.3389/fphar.2020.569489 (PMC7898238; doi:10.3389/fphar.2020.569489)
Supplement: Supplementary file 1 [file datasheet1.pdf]

Supplementary information

S1 Base model formulation

In this note, the formulation of the hiPSC-CM base model used in the simulations of the paper will be described. The model formulation can also be found in [1]. In the formulation, the membrane potential (v) is given in units of mV, and the Ca^{2+} concentrations are given in units of mM. Furthermore, the currents are given in units of μA/μF, and the Ca^{2+} fluxes are given in mmol/ms per total cell volume (i.e., in units of mM/ms). Time is expressed in ms. The parameters of the model are given in Tables S1–S6.

S1.1 Total membrane current

The total currents across the membrane is given by

$$\begin{aligned} I_{\mathrm{ion}} =& I_{\mathrm{Na}} + I_{\mathrm{NaL}} + I_{\mathrm{CaL}} + I_{\mathrm{to}} + I_{\mathrm{Kr}} + I_{\mathrm{Ks}} + I_{\mathrm{K1}} \\ &+ I_{\mathrm{NaCa}} + I_{\mathrm{NaK}} + I_{\mathrm{pCa}} + I_{\mathrm{bCl}} + I_{\mathrm{bCa}} + I_{\mathrm{f}}, \end{aligned} \tag{1}$$

where I_{Na}, I_{NaL}, I_{CaL}, I_{to}, I_{Kr}, I_{Ks}, I_{K1}, I_{NaCa}, I_{NaK}, I_{pCa}, I_{bCl}, I_{bCa}, and I_{f} are membrane currents that will be specified below.

In pure ODE simulations of the base model, the membrane potential is governed by

$$\frac{dv}{dt} = -(I_{\mathrm{ion}} + I_{\mathrm{stim}}), \tag{2}$$

where I_{stim} is an applied stimulus current. Note here that all the currents in I_{ion} are scaled by the specific membrane capacitance, C_m in units of μF/cm^2. Therefore, I_{ion} could be written as $I_{\mathrm{ion}} = \frac{1}{C_m}\tilde{I}_{\mathrm{ion}}$, where $\tilde{I}_{\mathrm{ion}}$ is given in units of μA/cm^2.

In bidomain-base model simulations, I_{ion} is included in the bidomain model

$$\chi C_m \left(\frac{\partial v}{\partial t} + I_{\text{ion}}(v, s) + I_{\text{stim}} \right) = \nabla \cdot (\sigma_i \nabla v) + \nabla \cdot (\sigma_i \nabla u_e), \tag{3}$$

$$0 = \nabla \cdot (\sigma_i \nabla v) + \nabla \cdot ((\sigma_i + \sigma_e) \nabla u_e), \tag{4}$$

$$\frac{\partial s}{\partial t} = F(v, s). \tag{5}$$

S1.2 Membrane currents

In general, the currents through the voltage-gated ion channels on the membrane are given on the form

$$I = go(v - E),$$

where g is the channel conductance, v is the membrane potential and E is the equilibrium potential of the channel. Moreover, $o = \prod_i z_i$ is the open probability of the channels, where z_i are gating variables, either given as an explicit function of v or governed by equations of the form

$$z_i' = \frac{1}{\tau_{z_i}} (z_{i,\infty} - z_i). \tag{6}$$

The parameters τ_{z_i} and $z_{i,\infty}$ for each of the gating variables of the base model are specified in Table S7.

S1.2.1 Fast sodium current

The formulation of the fast sodium current is based on the model in [2], adjusted to support slower upstroke velocities that are more similar to those observed in the optical measurements of hiPSC-CMs. The current is given by

$$I_{\text{Na}} = g_{\text{Na}} o_{\text{Na}} (v - E_{\text{Na}}). \tag{7}$$

Here, the open probability is given by

$$o_{\text{Na}} = m^3 j, \tag{8}$$

where m and j are gating variables governed by equations of the form (6).

S1.2.2 Late sodium current

The formulation of the late sodium current, I_{NaL}, is based on [3], and the current is given by

$$I_{\mathrm{NaL}} = g_{\mathrm{NaL}} o_{\mathrm{NaL}} (v - E_{\mathrm{Na}}). \tag{9}$$

Here, the open probability is given by

$$o_{\mathrm{NaL}} = m_L h_L, \tag{10}$$

where m_L and h_L are gating variables governed by equations of the form (6).

S1.2.3 Transient outward potassium current

The formulation of the transient outward potassium current, I_{to}, is based on [4], and the current is given by

$$I_{\mathrm{to}} = g_{\mathrm{to}} o_{\mathrm{to}} (v - E_{\mathrm{to}}). \tag{11}$$

Here, the open probability is given by

$$o_{\mathrm{to}} = q_{\mathrm{to}} r_{\mathrm{to}}, \tag{12}$$

where q_{to} and r_{to} are gating variables governed by equations of the form (6).

S1.2.4 Rapidly activating potassium current

The formulation of the rapidly activating potassium current, I_{Kr}, is based on [4], and the current is given by

$$I_{\mathrm{Kr}} = g_{\mathrm{Kr}} o_{\mathrm{Kr}} (v - E_{\mathrm{K}}). \tag{13}$$

Here,

$$o_{\mathrm{Kr}} = x_{\mathrm{Kr1}} x_{\mathrm{Kr2}}, \tag{14}$$

where the dynamics of x_{Kr1} and x_{Kr2} are governed by equations of the form (6).

S1.2.5 Slowly activating potassium current

The formulation of the slowly activating potassium current, I_{Ks}, is based on [2], and the current is given by

$$I_{\mathrm{Ks}} = g_{\mathrm{Ks}} o_{\mathrm{Ks}} (v - E_{\mathrm{Ks}}). \tag{15}$$

Here,

$$o_{\mathrm{Ks}} = x_{\mathrm{Ks}}^2, \tag{16}$$

where the dynamics of x_{Ks} is governed by an equation of the form (6).

S1.2.6 Inward rectifier potassium current

The formulation of the inward rectifier potassium current, I_{K1}, is based on [2], and the current is given by

$$I_{\mathrm{K1}} = g_{\mathrm{K1}} o_{\mathrm{K1}} (v - E_{\mathrm{K}}). \tag{17}$$

Here,

$$o_{\mathrm{K1}} = \frac{a_{\mathrm{K1}}}{a_{\mathrm{K1}} + b_{\mathrm{K1}}}, \tag{18}$$

$$a_{\mathrm{K1}} = \frac{1}{1 + e^{0.2(v - E_K - 59)}}, \tag{19}$$

$$b_{\mathrm{K1}} = \frac{0.5 e^{0.08(v - E_K + 5)} + e^{0.06(v - E_K - 594)}}{1 + e^{-0.5(v - E_K + 5)}}. \tag{20}$$

S1.2.7 Hyperpolarization activated funny current

The formulation for the hyperpolarization activated funny current, I_{f}, is based on [4], and the current is given by

$$I_{\mathrm{f}} = g_{\mathrm{f}} o_{\mathrm{f}} (v - E_{\mathrm{f}}). \tag{21}$$

Here,

$$o_{\mathrm{f}} = x_{\mathrm{f}}, \tag{22}$$

where the dynamics of x_{f} is governed by an equation of the form (6).

S1.2.8 L-type calcium current

The formualtion for the L-type calcium current, I_{CaL}, is based on the formulation in [2], and the current is given by

$$I_{\mathrm{CaL}} = g_{\mathrm{CaL}} o_{\mathrm{CaL}} \frac{(2F)^2 v}{RT} \frac{0.341 c_d e^{\frac{2Fv}{RT}} - 0.341 c_e}{e^{\frac{2Fv}{RT}} - 1}. \tag{23}$$

Here,

$$o_{\mathrm{CaL}} = df(1 - f_{\mathrm{Ca}}), \tag{24}$$

where the dynamics of d, f and f_{Ca} are governed by equations of the form (6).

S1.2.9 Background currents

The formulation of the background currents, I_{bCa} and I_{bCl}, are based on [2], and the currents are given by

$$I_{\mathrm{bCa}} = g_{\mathrm{bCa}}(v - E_{\mathrm{Ca}}), \tag{25}$$

$$I_{\mathrm{bCl}} = g_{\mathrm{bCl}}(v - E_{\mathrm{Cl}}). \tag{26}$$

S1.2.10 Sodium-calcium exchanger

The formulation of the sodium-calcium exchanger current, I_{NaCa}, is based on [2], and the current is given by

$$I_{\mathrm{NaCa}} = \bar{I}_{\mathrm{NaCa}} \frac{e^{\frac{\nu F v}{RT}}[\mathrm{Na}^+]_i^3 c_e - e^{\frac{(\nu-1)Fv}{RT}}[\mathrm{Na}^+]_e^3 c_{sl}}{s_{\mathrm{NaCa}}\left(1 + \left(\frac{K_{\mathrm{act}}}{c_{sl}}\right)^2\right)\left(1 + k_{\mathrm{sat}} e^{\frac{(\nu-1)Fv}{RT}}\right)}. \tag{27}$$

Here,

$$\begin{aligned} s_{\mathrm{NaCa}} = {} & K_{\mathrm{Ca},i}[\mathrm{Na}^+]_e^3 \left(1 + \left(\frac{[\mathrm{Na}^+]_i}{K_{\mathrm{Na},i}}\right)^3\right) + K_{\mathrm{Na},e}^3 c_{sl}\left(1 + \frac{c_{sl}}{K_{\mathrm{Ca},i}}\right) \\ & + K_{\mathrm{Ca},e}[\mathrm{Na}^+]_i^3 + [\mathrm{Na}^+]_i^3 c_e + [\mathrm{Na}^+]_e^3 c_{sl}. \end{aligned}$$

S1.2.11 Sarcolemmal calcium pump

The formulation of the current through the sarcolemmal calcium pump, I_{pCa}, is based on [2], and the current is given by

$$I_{\mathrm{pCa}} = \bar{I}_{\mathrm{pCa}} \frac{c_{sl}^2}{K_{\mathrm{pCa}}^2 + c_{sl}^2}. \tag{28}$$

S1.2.12 Sodium-potassium pump

The current through the sodium-potassium pump, I_{NaK}, is based on [2], and the current is given by

$$I_{\mathrm{NaK}} = \bar{I}_{\mathrm{NaK}} \frac{f_{\mathrm{NaK}}}{1 + \left(\frac{K_{\mathrm{Na,i}}^{\mathrm{NaK}}}{[\mathrm{Na}^+]_{\mathrm{i}}}\right)^4} \frac{[\mathrm{K}^+]_{\mathrm{e}}}{[\mathrm{K}^+]_{\mathrm{e}} + K_{\mathrm{K,e}}}. \tag{29}$$

Here,

$$f_{\mathrm{NaK}} = \frac{1}{1 + 0.12 e^{-0.1\frac{Fv}{RT}}} + \frac{0.037}{7}\left(e^{\frac{[\mathrm{Na}^+]_{\mathrm{e}}}{67}} - 1\right) e^{-\frac{Fv}{RT}}. \tag{30}$$

S1.3 Calcium dynamics

The calcium dynamics of the base model are governed by the following system of equations:

$$\frac{dc_d}{dt} = \frac{1}{V_d}(J_{\mathrm{CaL}} - J_d^b - J_d^c), \qquad \frac{db_d}{dt} = \frac{1}{V_d}J_d^b, \tag{31}$$

$$\frac{dc_{sl}}{dt} = \frac{1}{V_{sl}}(J_e^{sl} - J_{sl}^c - J_{sl}^b + J_s^{sl}), \qquad \frac{db_{sl}}{dt} = \frac{1}{V_{sl}}J_{sl}^b, \tag{32}$$

$$\frac{dc_c}{dt} = \frac{1}{V_c}(J_{sl}^c + J_d^c - J_c^n - J_c^b), \qquad \frac{db_c}{dt} = \frac{1}{V_c}J_c^b, \tag{33}$$

$$\frac{dc_s}{dt} = \frac{1}{V_s}(J_n^s - J_s^{sl} - J_s^b), \qquad \frac{db_s}{dt} = \frac{1}{V_s}J_s^b, \tag{34}$$

$$\frac{dc_n}{dt} = \frac{1}{V_n}(J_c^n - J_n^s). \tag{35}$$

Here, c_d is the concentration of free calcium in the dyad, b_d is the concentration of calcium bound to a buffer in the dyad, c_{sl} is the concentration of free calcium in the sub-sarcolemmal (SL) compartment, b_{sl} is the concentration of calcium bound to a buffer in the SL compartment, c_c is the concentration of free calcium in the bulk cytosol, b_c is the concentration of calcium bound to a buffer in the bulk cytosol, c_s is the concentration of free calcium in the junctional sarcoplasmic reticulum (jSR) compartment, b_s is the concentration of calcium bound to a buffer in the jSR, and c_n is the concentration of free calcium in the network sarcoplasmic reticulum (nSR) compartment. Below, each of the calcium fluxes will be specified.

S1.3.1 Flux through the SERCA pumps

The formulation of the flux from the bulk cytosol to the nSR through the SERCA pumps is based on [2] and is expressed by

$$J_c^n = \bar{J}_{\mathrm{SERCA}} \frac{\left(\frac{c_c}{K_c}\right)^2 - \left(\frac{c_n}{K_n}\right)^2}{1 + \left(\frac{c_c}{K_c}\right)^2 + \left(\frac{c_n}{K_n}\right)^2}. \tag{36}$$

Flux through the RyRs The flux from the jSR to the SL compartment is given by

$$J_s^{sl} = J_{\mathrm{RyR}} + J_{\mathrm{leak}}, \tag{37}$$

where J_{RyR} is flux through active RyR channels and J_{leak} is flux through RyR channels that are always open, given by

$$J_{\mathrm{RyR}} = p \cdot r \cdot \alpha_{\mathrm{RyR}}(c_s - c_{sl}), \tag{38}$$

$$J_{\mathrm{leak}} = \gamma_{\mathrm{RyR}} \cdot \alpha_{\mathrm{RyR}}(c_s - c_{sl}), \tag{39}$$

respectively. Here, p represents the open probability of the active RyR channels, and this is given by

$$p = \frac{c_d^3}{c_d^3 + \kappa_{\mathrm{RyR}}^3}. \tag{40}$$

Furthermore, r is the fraction of RyR channels that are not inactivated and is governed by the equation

$$\frac{dr}{dt} = -\frac{J_{\mathrm{RyR}}}{\beta_{\mathrm{RyR}}} + \frac{\eta_{\mathrm{RyR}}}{p}(1 - r). \tag{41}$$

See [1] for a derivation of these expressions.

S1.3.2 Passive diffusion fluxes between compartments

The formulation of the passive diffusion fluxes between compartments are based on e.g. [2], and the fluxes are given by

$$J_d^c = \alpha_d^c(c_d - c_c), \tag{42}$$

$$J_{sl}^c = \alpha_{sl}^c(c_{sl} - c_c), \tag{43}$$

$$J_n^s = \alpha_n^s(c_n - c_s). \tag{44}$$

S1.3.3 Buffer fluxes

The formulation of the fluxes of free calcium binding to a calcium buffer are based on e.g. [2], and the fluxes are given by

$$J_d^b = V_d(k_{\mathrm{on}}^d c_d(B_{\mathrm{tot}}^d - b_d) - k_{\mathrm{off}}^d b_d), \tag{45}$$

$$J_{sl}^b = V_{sl}(k_{\mathrm{on}}^{sl} c_{sl}(B_{\mathrm{tot}}^{sl} - b_{sl}) - k_{\mathrm{off}}^{sl} b_{sl}), \tag{46}$$

$$J_c^b = V_c(k_{\mathrm{on}}^c c_c(B_{\mathrm{tot}}^c - b_c) - k_{\mathrm{off}}^c b_c), \tag{47}$$

$$J_s^b = V_s(k_{\mathrm{on}}^s c_s(B_{\mathrm{tot}}^s - b_s) - k_{\mathrm{off}}^s b_s). \tag{48}$$

S1.3.4 Membrane fluxes

The membrane fluxes, J_{CaL}, J_{bCa}, J_{pCa}, and J_{NaCa}, are given by

$$J_{\mathrm{CaL}} = -\frac{\chi C_m}{2F} I_{\mathrm{CaL}}, \qquad J_{\mathrm{pCa}} = -\frac{\chi C_m}{2F} I_{\mathrm{pCa}}, \tag{49}$$

$$J_{\mathrm{bCa}} = -\frac{\chi C_m}{2F} I_{\mathrm{bCa}}, \qquad J_{\mathrm{NaCa}} = \frac{\chi C_m}{F} I_{\mathrm{NaCa}}, \tag{50}$$

(see [1]). Here, I_{CaL}, I_{bCa}, I_{pCa}, and I_{NaCa} are defined by the expressions given above. Note also that J_e^{sl} is defined by

$$J_e^{sl} = J_{\mathrm{NaCa}} + J_{\mathrm{pCa}} + J_{\mathrm{bCa}}. \tag{51}$$

S1.4 Nernst equilibrium potentials

The Nernst equilibrium potentials for the different types of ion channels are defined as

$$E_{\mathrm{Na}} = \frac{RT}{F} \log\left(\frac{[\mathrm{Na}^+]_{\mathrm{e}}}{[\mathrm{Na}^+]_{\mathrm{i}}}\right), \tag{52}$$

$$E_{\mathrm{Ca}} = \frac{RT}{2F} \log\left(\frac{[\mathrm{Ca}^{2+}]_{\mathrm{e}}}{c_{sl}}\right), \tag{53}$$

$$E_{\mathrm{K}} = \frac{RT}{F} \log\left(\frac{[\mathrm{K}^+]_{\mathrm{e}}}{[\mathrm{K}^+]_{\mathrm{i}}}\right), \tag{54}$$

$$E_{\mathrm{Ks}} = \frac{RT}{F} \log\left(\frac{[\mathrm{K}^+]_{\mathrm{e}} + 0.018[\mathrm{Na}^+]_{\mathrm{e}}}{[\mathrm{K}^+]_{\mathrm{i}} + 0.018[\mathrm{Na}^+]_{\mathrm{i}}}\right), \tag{55}$$

$$E_{\mathrm{Cl}} = \frac{RT}{F} \log\left(\frac{[\mathrm{Cl}^+]_{\mathrm{e}}}{[\mathrm{Cl}^+]_{\mathrm{i}}}\right), \tag{56}$$

$$E_{\mathrm{f}} = -17 \text{ mV}, \tag{57}$$

for the parameter values given in Table S2.

Parameter	Description	Value
V_d	Volume fraction of the dyadic subspace	0.001
V_{sl}	Volume fraction of the SL compartment	0.028
V_c	Volume fraction of the bulk cytosol	0.917
V_s	Volume fraction of the jSR	0.004
V_n	Volume fraction of the nSR	0.05
χ	Cell surface to volume ratio	0.9 μm^{-1}

Table S1: Geometry parameters of the hiPSC-CM base model.

Parameter	Description	Value
C_m	Specific membrane capacitance	0.01 μF/μm^2
F	Faraday's constant	96.485 C/mmol
R	Universal gas constant	8.314 J/(mol·K)
T	Temperature	310 K
$[Ca^{2+}]_e$	Extracellular Ca^{2+} concentration	0.42 mM
$[Na^+]_e$	Extracellular sodium concentration	140 mM
$[Na^+]_i$	Intracellular sodium concentration	8 mM
$[K^+]_e$	Extracellular potassium concentration	5 mM
$[K^+]_i$	Intracellular potassium concentration	120 mM
$[Cl^-]_e$	Extracellular chloride concentration	150 mM
$[Cl^-]_i$	Intracellular chloride concentration	15 mM

Table S2: Physical constants and ionic concentrations of the base model.

Parameter	Value	Parameter	Value
g_{Na}	2.6 mS/μF	g_{CaL}	1.8 nL/(μF ms)
g_{NaL}	0.03 mS/μF	g_{bCa}	0.0005 mS/μF
g_{to}	0.21 mS/μF	$\bar{I}_{\text{NaCa}}$	9.4 μA/μF
g_{Kr}	0.075 mS/μF	$\bar{I}_{\text{pCa}}$	0.12 μA/μF
g_{Ks}	0.0127 mS/μF	$\bar{J}_{\text{SERCA}}$	0.00016 mM/ms
g_{K1}	0.05 mS/μF	α_{RyR}	0.0052 ms^{-1}
g_{f}	0.012 mS/μF	α_d^c	0.0027 ms^{-1}
g_{bCl}	0.0001 mS/μF	α_{sl}^c	0.3 ms^{-1}
$\bar{I}_{\text{NaK}}$	1.9 μA/μF	α_n^s	0.0093 ms^{-1}

Table S3: Conductance values and similar parameters for each of the membrane currents and intracellular calcium fluxes of the hiPSC-CM base model.

Parameter	Flux	Value
K_c	J_c^n	0.00025 mM
K_n	J_c^n	1.7 mM
β_{RyR}	J_s^{sl}	0.0265 mM
γ_{RyR}	J_s^{sl}	0.001
κ_{RyR}	J_{RyR}	0.015 mM
η_{RyR}	J_s^{sl}	0.00001 ms^{-1}

Table S4: Remaining parameters for the intracellular calcium fluxes of the base model.

Parameter	Current	Value
k_{sat}	I_{NaCa}	0.3
ν	I_{NaCa}	0.3
K_{act}	I_{NaCa}	0.00015 mM
$K_{\text{Ca},i}$	I_{NaCa}	0.0036 mM
$K_{\text{Ca},e}$	I_{NaCa}	1.3 mM
$K_{\text{Na},i}$	I_{NaCa}	12.3 mM
$K_{\text{Na},e}$	I_{NaCa}	87.5 mM
$K_{\text{Na},i}^{\text{NaK}}$	I_{NaK}	11 mM
$K_{\text{K},e}$	I_{NaK}	1.5 mM
K_{pCa}	I_{pCa}	0.0005 mM

Table S5: Remaining parameters for the membrane currents of the base model.

Parameter	Compartment	Value
B_{tot}^{c}	Bulk cytosol	0.063 mM
k_{on}^{c}	Bulk cytosol	40 $\text{ms}^{-1}\text{mM}^{-1}$
k_{off}^{c}	Bulk cytosol	0.03 ms^{-1}
B_{tot}^{d}	Dyad	2.7 mM
k_{on}^{d}	Dyad	100 $\text{ms}^{-1}\text{mM}^{-1}$
k_{off}^{d}	Dyad	1 ms^{-1}
B_{tot}^{sl}	Subsarcolemmal space	1.45 mM
k_{on}^{sl}	Subsarcolemmal space	100 $\text{ms}^{-1}\text{mM}^{-1}$
k_{off}^{sl}	Subsarcolemmal space	0.15 ms^{-1}
B_{tot}^{s}	Junctional SR	60 mM
k_{on}^{s}	Junctional SR	100 $\text{ms}^{-1}\text{mM}^{-1}$
k_{off}^{s}	Junctional SR	65 ms^{-1}

Table S6: Parameters for the Ca^{2+} buffers of the base model.

Current	Gate	z_∞	α_z	β_z	τ_z
I_{Na}	m	$\frac{1}{(1+e^{(-57-v)/9})^2}$	$0.13e^{-((v+46)/16)^2}$	$0.06e^{-((v-5)/51)^2}$	$\alpha_m+\beta_m$
	j	$\frac{1}{(1+e^{(v+72)/7})^2}$	$\begin{cases} 0, & \text{if } v \geq -40 \\ \frac{\begin{pmatrix} -2.5\cdot 10^4 e^{0.2v} \\ -7\cdot 10^{-6}e^{-0.04v} \end{pmatrix}(v+38)}{1+e^{0.3(v+79)}}, & \text{otherwise} \end{cases}$	$\begin{cases} \frac{0.6e^{0.06v}}{1+e^{-0.1(v+32)}}, & \text{if } v \geq -40 \\ \frac{0.02e^{-0.01v}}{1+e^{-0.14(v+40)}}, & \text{otherwise} \end{cases}$	$\frac{1}{\alpha_j+\beta_j}$
I_{NaL}	m_L	$\frac{1}{1+e^{(-43-v)/5}}$	$\frac{1}{6.8e^{(v+12)/35}}$	$8.6e^{-(v+77)/6}$	$\alpha_m+\beta_m$
	h_L	$\frac{1}{1+e^{(v+88)/7.5}}$			200 ms
I_{CaL}	d	$\frac{1}{1+e^{-(v+5)/6}}$	$\frac{1-e^{-\frac{v+5}{6}}}{0.035(v+5)}$		$\alpha_d d_\infty$
	f	$\frac{1}{1+e^{(v+35)/9}}+\frac{0.6}{1+e^{(50-v)/20}}$	$\frac{1}{0.02e^{-(0.034(v+14.5)^2)}+0.02}$		α_f
	f_{Ca}	$\frac{1.7c_d}{1.7c_d+0.012}$	$\frac{1}{1.7c_d+0.012}$		α_{Ca}
I_{to}	q_{to}	$\frac{1}{1+e^{(v+53)/13}}$	$\frac{39}{0.57e^{-0.08(v+44)}+0.065e^{0.1(v+46)}}$	6	$\alpha_{q_{\text{to}}}+\beta_{q_{\text{to}}}$
	r_{to}	$\frac{1}{1+e^{-(v-22.3)/18.75}}$	$\frac{14.4}{e^{0.09(v+30.61)}+0.37e^{-0.12(v+24)}}$	2.75	$\alpha_{r_{\text{to}}}+\beta_{r_{\text{to}}}$
I_{Kr}	x_{Kr1}	$\frac{1}{1+e^{-(v+20.7)/4.9}}$	$\frac{450}{1+e^{-(v+45)/10}}$	$\frac{6}{1+e^{(v+30)/11.5}}$	$\alpha_{x_{\text{Kr1}}}\cdot\beta_{x_{\text{Kr1}}}$
	x_{Kr2}	$\frac{1}{1+e^{(v+88)/50}}$	$\frac{3}{1+e^{-(v+60)/20}}$	$\frac{1.12}{1+e^{(v-60)/20}}$	$\alpha_{x_{\text{Kr2}}}\cdot\beta_{x_{\text{Kr2}}}$
I_{Ks}	x_{Ks}	$\frac{1}{1+e^{-(v+3.8)/14}}$	$\frac{990}{1+e^{-(v+2.4)/14}}$		$\alpha_{x_{\text{Ks}}}$
I_{f}	x_{f}	$\frac{1}{1+e^{(v+78)/5}}$	$\frac{1900}{1+e^{(v+15)/10}}$		$\alpha_{x_{\text{Ks}}}$

Table S7: Specification of the parameters z_∞ and τ_z, for $z = m$, j, m_L, h_L, d, f, f_{Ca}, q_{to}, r_{to}, x_{Kr1}, x_{Kr2}, x_{Ks} and x_{f} in the equations for the gating variables of the form (6).

References

[1] Karoline Horgmo Jæger, Verena Charwat, Bérénice Charrez, Henrik Finsberg, Mary M Maleckar, Sam Wall, Kevin Healy, and Aslak Tveito. Improved computational identification of drug response using optical measurements of human stem cell derived cardiomyocytes in microphysiological systems. *bioRxiv*, page 787390, 2019.

[2] Eleonora Grandi, Francesco S Pasqualini, and Donald M Bers. A novel computational model of the human ventricular action potential and Ca transient. *Journal of Molecular and Cellular Cardiology*, 48(1):112–121, 2010.

[3] Thomas O'Hara, László Virág, András Varró, and Yoram Rudy. Simulation of the undiseased human cardiac ventricular action potential: Model formulation and experimental validation. *PLoS Computational Biology*, 7(5):e1002061, 2011.

[4] Michelangelo Paci, Jari Hyttinen, Katriina Aalto-Setälä, and Stefano Severi. Computational models of ventricular-and atrial-like human induced pluripotent stem cell derived cardiomyocytes. *Annals of Biomedical Engineering*, 41(11):2334–2348, 2013.
